# Accuracy of reticulocyte hemoglobin for diagnosing iron deficiency in former very preterm infants: a population-based cohort study

**DOI:** 10.3389/fped.2023.1281513

**Published:** 2023-11-20

**Authors:** Hudson Barr, Ketan Kulkarni, Balpreet Singh, Navjot Sandila, Lisa Morrison, Lori Beach, Satvinder Ghotra

**Affiliations:** ^1^ Department of Pediatrics, IWK Health Centre, Halifax, NS, Canada; ^2^ Faculty of Medicine, Dalhousie University, Halifax, NS, Canada; ^3^ Research Methods Unit, Nova Scotia Health Authority, Halifax, NS, Canada

**Keywords:** iron deficiency, preterm, reticulocyte hemoglobin, serum ferritin, accuracy

## Abstract

**Background:**

Serum ferritin (SF) is commonly used to diagnose iron deficiency (ID) but has limitations. Reticulocyte hemoglobin (Ret-He) is being increasingly used for ID diagnosis. This study aimed to assess accuracy of Ret-He for ID diagnosis in former very preterm infants (VPI) at 4–6 months corrected age (CA).

**Methods:**

A retrospective population-based cohort study was conducted on all live VPI born between 23 and 30 weeks of gestational age (GA) in Nova Scotia from 2012 to 2018. Infants underwent SF and Ret-He testing at 4–6 months CA. ID was defined using two definitions. The first defined ID as SF < 20 mcg/L at both 4- and 6-months CA, and the second as SF < 30 mcg at at both 4- and 6-months CA. The accuracy of Ret-He for identifying ID was assessed using the area under the receiver operating characteristic curve (AUC).

**Results:**

ID was present in 39.7% (62) of 156 infants in the first definition and 59.6% (93) in the second at 4–6 months CA. The AUC of Ret-He for ID diagnosis was 0.64 (*p* = 0.002) in the first definition and 0.59 (*p* = 0.04) in the second. The optimal cut-off was 29.4pg in the first and 29.7 in the second definition. The sensitivity, specificity, positive predictive value (PPV), and negative predictive values (NPV) at the 29.4 pg cut-off were 50.0%, 78.7%, 60.8%, and 70.5% for definition 1 and 44.1%, 74.6%, 71.9%, and 47.5% at the 29.7pg cut-off for definition 2.

**Conclusion:**

Ret-He had low diagnostic accuracy for ID diagnosis in former VPI. Caution is advised when using Ret-He alone for ID diagnosis. Further research is needed to establish optimal approaches for identifying ID in VPI.

## 1. Introduction

Iron deficiency (ID) is common worldwide and is also identified as a public health issue in Canadian infants and young children (1). Infants born prematurely have an increased risk of developing ID (2). In a recent study (3), authors found that 32% of very preterm infants (VPI) develop ID by 6-months of age, despite preventative iron supplementation. ID leading to anemia during childhood is associated with long-term impairments in motor, cognitive, socioemotional, and neurophysiologic development, and even without anemia, ID has been linked to similar adverse cognitive and behavioral outcomes (4). Research suggests that the adverse effects of ID on infants’ brains are irreversible since later iron supplementation may not improve impairments caused by ID in these infants (5). Therefore, preventing and diagnosing ID early in this population is important to avoid negative neurodevelopmental consequences (3).

Serum ferritin (SF) is the most widely used test for diagnosing ID, and it is also the earliest marker of ID, as its concentration is proportional to the body’s total iron stores (6). However, there are limitations to using SF as a reliable indicator of ID. SF is an acute-phase protein that can be affected by infection and inflammation, making it difficult to accurately reflect the iron stores in the body in certain situations. Moreover, as SF tests can be quite expensive in developing countries, there has been increasing interest in alternative tests for ID.

Another ID marker that has gained more popularity since the mid 2000s is reticulocyte hemoglobin equivalent (Ret-He) (7). Ret-He is a measure of the iron content in circulating reticulocytes, which are newly released red blood cells from the bone marrow, and has the advantage of providing an immediate measure of iron availability in the body in as little as 2 min (8). Additionally, Ret-He tests are fully automated and can be performed on the same specimen as for a complete blood count (CBC), whereas SF measurements require an additional sample of blood as it is a serum sample (8). Furthermore, Ret-He is not affected by inflammation and infection. The above reasons have led to the increased use of Ret-He for diagnosing ID in several pediatric populations (9–11). It is also increasingly reported in preterm infants during the first few weeks of life (11, 12). However, there is a lack of literature on use of Ret-He as a reliable ID marker in preterm infants during the first year of life. The objective of the current study aims to assess the diagnostic accuracy of Ret-He as an ID indicator for preterm infants at 4–6 months of age when compared against the SF levels.

## 2. Materials and methods

### 2.1. Study design and participants

This retrospective population-based cohort study of diagnostic accuracy included all live-born VPI born between 23 and 30 weeks gestational age (GA) from 2012 to 2018 in the province of Nova Scotia. STARD Guidelines (Updated 2015) were used as the study model (13). Exclusion criteria included infants with hematological disorders and chromosomal or major congenital anomalies.

As per Canadian Pediatric Society guidelines, all patients were provided with prophylactic iron supplementation of 2–4 mg/kg/day beginning at 2–4 weeks of chronological age (14, 15).

Iron dose was further regularly titrated during the hospital stay based on hemoglobin concentration, reticulocyte count, SF and Ret-He levels. Starting in 2012, Ret-He was used as a marker to guide iron requirements, especially when SF levels were not available or unreliable in the presence of an acute infection/inflammation or recent blood transfusion. Erythropoietic stimulants were not used at the study hospital. Iron prophylaxis was recommended until 9–12 months corrected age (CA). All infants were seen through the provincial perinatal follow-up program (PFUP) at 4- or 6-months CA for a growth and neurodevelopmental check. Follow-up at 4 months was performed for infants if they are at a higher risk for growth or neurodevelopmental issues (such as born at 23–28 weeks GA or had major brain injury), while a 6-month follow-up was arranged if the infant was considered low risk (29–30 weeks GA or no major brain injury). At 4–6 months CA, CBC and SF testing was also performed on all infants to check iron stores and guide iron supplementation. In 2012, Ret-He was added to blood work as an additional marker of ID and was considered for clinical decision-making in conjunction with other ID markers.

The CBC and reticulocyte parameters were measured with a Sysmex XE-5000 hematology analyzer. Ferritin was measured on a Beckman UniCel DxI 800 hematology analyzer. Clinical information and the results of SF testing were not available to the performers or readers of the Ret-He test. Similarly, clinical information and Ret-He test results were not available to the assessors of the SF test.

All relevant data was retrieved from the population-based AC Allen PFUP database and electronic chart reviews. This study performed two analyses. For the main analysis (definition 1) in this study, ID was defined as SF level <20 mcg/L at both 4 and 6 months CA, as completed as standard practice by the present study hospital’s laboratory. As a secondary analysis (definition 2), ID was defined using SF level <30 mcg/L at both 4 and 6 months CA, as recommended by a recent modified Delphi study for preterm infants (16). The study was approved by the Research Ethics Board of the IWK Health (Approval 1026862). The Research Ethics Board approved a waiver of informed consent.

### 2.2. Statistical analysis

Baseline characteristics were summarized as mean (sd) for continuous variables and as frequency (percent) for categorical variables. Mean differences and odds ratios along with 95% confidence intervals of the baseline characteristics were also calculated. To assess the accuracy of Ret-He for correctly classifying patients with and without ID, receiver operating characteristic (ROC) curves were performed and the area under the curves (AUC) were calculated using both definitions of ID. The AUC of a diagnostic test must be greater than 0.5 for it to have a diagnostic ability greater than random chance, with an AUC ≥ 0.8 generally considered acceptable and an AUC ≤ 0.70 considered poor. The optimal cut-off point for Ret-He was determined by maximizing the Youden’s index (sensitivity + specificity −1). This point corresponds to the point on the ROC curve with the greatest vertical distance from the diagonal line, which is the ROC of a test with no discriminatory ability. The sensitivity, specificity, positive predictive value (PPV), and negative predictive value (NPV) at this cut-off point were calculated. As an exploratory analysis, AUC was also estimated after adjusting for GA and birth weight. The Spearman correlation coefficient between SF and Ret-He was calculated. A two-sided *P* value of < 0.05 was the threshold for statistical significance. Participants with indeterminate or missing data for either Ret-He or SF were removed from analysis. All analyses were performed using SAS statistical software version 9.4 (SAS Institute Inc., Cary, N.C, USA).

## 3. Results

### 3.1. Patient characteristics

A total of 156 patients were included in the study. In the main analysis, 62 (39.7%) met the criteria for ID diagnosis at 4–6 months CA. The mean levels were lower for patients in the ID group compared to the non-ID group for Ret-He, SF, and mean corpuscular hemoglobin concentration (MCHC). Additional information such as the baseline characteristics of patients, and other iron indices, are presented in Table 1. In the secondary analysis, 59.6% (93) met the criteria for ID diagnosis at 4–6 months CA. The mean levels were lower for patients in the ID group compared to non-ID group for SF and Ret-HeAdditional information such as the baseline characteristics of patients, and other iron indices, can be found in Table 2.

**Table 1:** Baseline characteristics of the iron-deficient and Non-iron deficient study population as defined by SF < 20 mcg/L at 4–6 months corrected age.

Characteristic	Iron Deficiency Group *N* = 62 *N* (%)	Non-Iron deficiency group *N* = 94 *N* (%)	OR or mean difference (95% CI)
NICU Variables			
Mean gestational Age, weeks, ±SD	28.2 ± 1.7	28.1 ± 1.8	−0.1 (−0.7 to 0.5)
Gestational Age, weeks			
23–26	10 (16.1)	19 (20.2)	0.8 (0.3 to 1.8)
27–30	52 (83.4)	75 (79.8)	ref
Mean birth weight, grams, ±SD	1173.8 ± 305.3	1197.1 ± 325.1	23.3 (−79.2 to 126.0)
Male sex	28 (45.2)	55 (58.5)	0.6 (0.3 to 1.1)
Mean ferritin at discharge, µg/L, ±SD	83.7 ± 74.2	86.7 ± 69.4	3.0 (−25.0 to 31.0)
Mean iron dose at discharge, mg/kg/d, ±SD*	2.6 ± 0.9	2.9 ± 0.9	0.3 (0.02 to 0.6)
Received RBC Transfusion	31 (49.2)	43 (46.4)	1.1 (0.6 to 2.1)
At Follow Up			
Mean corrected age, months, ±SD	5.1 ± 1.2	5.2 ± 1.1	0.1 (−0.3 to 0.4)
Lab Markers at Follow-Up, mean ± SD			
Ferritin, µg/L*	15.0 ± 5.0	45.7 ± 29.5	30.7 (23.2 to 38.2)
MCV, fL	78.5 ± 3.8	78.9 ± 3.2	0.4 (−0.7 to 1.6)
MCH, pg	27.0 ± 1.6	27.5 ± 1.2	0.5 (−0.1 to 1.0)
MCHC, g/L*	343.4 ± 10.1	348.6 ± 9.5	5.1 (2.0 to 8.3)
RDW, %	12.9 ± 0.9	12.7 ± 0.7	−0.2 (−0.5 to 0.005)
RetCount, %	1.1 ± 0.3	1.2 ± 0.4	0.0 (−0.1 to 0.2)
Ret-He, pg*	29.6 ± 2.7	30.9 ± 2.2	1.3 (0.5 to 2.1)
Hemoglobin, g/L	121.1 ± 10.5	122.7 ± 9.4	1.6 (−1.6 to 4.7)

SD, standard deviation; RBC, red blood cell; MCV, mean corpuscular volume; MCH, mean corpuscular hemoglobin; MCHC, mean corpuscular hemoglobin concentration; RDW, red cell distribution width; RetCount, reticulocyte count; Ret-He, Reticulocyte Hemoglobin equivalent.

* Denotes *p* < 0.05.

**Table 2:** Baseline characteristics of the iron-deficient and Non-iron deficient study population as defined by SF < 30 mcg/l at 4–6 months corrected age.

Characteristic	Iron Deficiency Group *N* = 93 *N* (%)	Non-Iron deficiency group *N* = 63 *N* (%)	OR or mean difference (95% CI)
NICU Variables			
Mean gestational Age, weeks, ±SD	28.1 ± 1.7	28.1 ± 1.8	0.0 (−0.5 to 0.6)
Gestational Age, weeks			
23–26	18 (19.4)	11 (17.5)	1.1 (0.5 to 2.6)
27–30	75 (80.6)	52 (82.5)	ref
Mean birth weight, grams, ±SD	1177.8 ± 334.5	1202.7 ± 290.0	24.8 (−77.5 to 127.0)
Male sex	48 (51.6)	35 (55.6)	0.9 (0.4 to 1.6)
Mean ferritin at discharge, µg/L, ±SD	81.2 ± 65.6	92.8 ± 79.9	11.6 (−16.9 to 40.0)
Mean iron dose at discharge, mg/kg/d, ±SD	2.6 ± 1.0	2.9 ± 0.8	0.3 (−0.3 to 0.6)
Received RBC Transfusion	46 (49.5)	28 (44.4)	1.2 (0.6 to 2.2)
At Follow-Up			
Mean corrected age, months, ±SD	5.1 ± 1.0	5.3 ± 1.2	0.2 (−0.2 to 0.6)
Lab Markers at Follow-Up, mean ± SD			
Ferritin, µg/L*	17.9 ± 6.1	56.6 ± 30.1	38.7 (32.3 to 45.1)
MCV, fL	78.6 ± 3.5	79.0 ± 3.4	0.4 (−0.7 to 1.5)
MCH, pg	27.2 ± 1.4	27.5 ± 1.4	0.3 (−0.2 to 0.7)
MCHC, g/L	345.8 ± 10.2	347.6 ± 9.9	1.7 (−1.5 to 5.0)
RDW, %	12.8 ± 0.9	12.7 ± 0.6	−0.1 (−0.4 to 0.1)
RetCount, %	1.1 ± 0.3	1.2 ± 0.4	0.1 (−0.1 to 0.2)
Ret-He, pg*	30.0 ± 2.5	30.9 ± 2.3	0.9 (0.1 to 1.6)
Hemoglobin, g/L	121.8 ± 9.63	122.4 ± 10.2	0.5 (−2.6 to 3.7)

SD, standard deviation; RBC, red blood cell; MCV, mean corpuscular volume; MCH, mean corpuscular hemoglobin; MCHC, mean corpuscular hemoglobin concentration; RDW, red cell distribution width; RetCount, reticulocyte count; Ret-He, reticulocyte hemoglobin equivalent.

* Denotes *p* < 0.05.

### 3.2. ROC & Cut-offs

**Figure 1:**
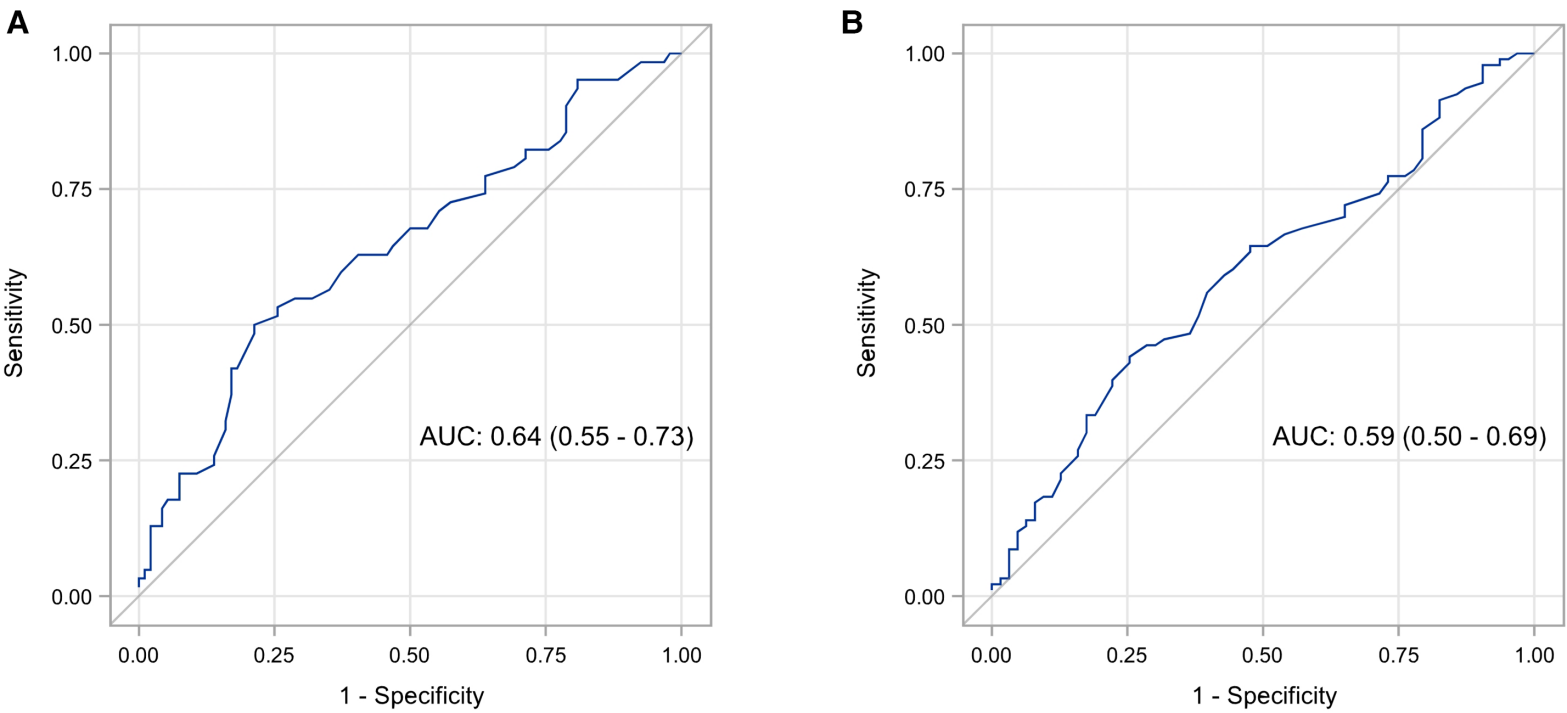
ROC analysis of Ret-He for diagnosis of iron deficiency in premature infants using two definitions: serum ferritin levels <20 mcg/L at 4–6 months corrected age (**A**), and serum ferritin levels <30 mcg/L at 4–6 months corrected age (**B**).

Spearman correlation coefficient between SF and Ret-He was 0.23 (95% CI: 0.08–0.37, *p* = 0.004). In definition 1, the AUC of Ret-He for the diagnosis of ID was 0.64 (95% CI: 0.55–0.73, *p* = 0.002) (shown in Figure 1A). The optimal cut-off value of Ret-He for ID diagnosis was established at 29.4 pg. Sensitivity, specificity, PPV, and NPV were 50.0%, 78.7%, 60.8%, and 70.5%, respectively (Table 3). The AUC of the model did not change significantly when other predictors like birth weight and GA were added (95% CI: −0.06–0.02, *p* = 0.355). In definition 2, the AUC of Ret-He for ID diagnosis was 0.59 (95% CI: 0.50–0.68, *p* = 0.04) (shown in Figure 1B). The optimal cut-off value of Ret-He for ID diagnosis was established at 29.7 pg. Sensitivity, specificity, PPV, and NPV were 44.1%, 74.6%, 71.9%, and 47.5%, respectively (Table 3).

**Table 3:** Predictive ability of Ret-He for diagnosing iron deficiency using two different definitions of iron deficiency.

Ret-He	Definition 1 SF level <20 mcg/L at 4 or 6 months	Definition 2 SF level <30 mcg/L at 4 or 6 months
Cut-off (pg)	29.4	29.7
AUROC (95% CI)	0.64 (0.55, 0.73)	0.59 (0.50, 0.68)
Sensitivity (%)	50.0 (37. 6, 62.4)	44.1 (33.8, 54.8)
Specificity (%)	78.7 (70.4, 87.0)	74.6 (62.1, 84.7)
Youden’s Index	0.29	0.19
PPV (%)	60.8 (47.4, 74.2)	71.9 (58.5, 83.0)
NPV (%)	70.5 (61.8, 79.2)	47.5 (37.3, 57.8)
*p*-value	0.002	0.041

AUROC, area under receiver operator characteristics; CI, confidence interval; PPV, positive predictive value; NPV, negative predictive value. In definition 1, ID was defined as SF level <20 mcg/L at 4 or 6 months. In definition 2, ID was defined as SF level <30 mcg/L at 4–6 months corrected age.

## 4. Discussion

This population-based cohort study assessed the utility of Ret-He for diagnosing ID compared to SF in former VPI. ID patients had lower Ret-He and SF levels than did non-ID patients. The AUC of Ret-He for ID diagnosis ranged from 0.59 to 0.64, depending on the SF cut-off used. The optimal cut-off value of Ret-He for ID diagnosis was established as 29.4 pg or 29.7 pg depending upon the cut-off used to define ID. Sensitivity, specificity, PPV, and NPV also varied depending on the ID definition.

Ret-He has been extensively studied to diagnose ID in older pediatric and adult populations. However, its utility in infant populations is less known. Given how rapidly infants grow, it may be inappropriate to extrapolate cut-off values from other patient populations. Additionally, appropriate cut-off values for Ret-He vary depending on the age and other clinical factors. For instance, Brugnara found a reticulocyte hemoglobin content (CHr) cutoff of 26 pg in the diagnosis of ID in young children averaging 2.9 years of age (17), while Ullrich et al. (18) showed that CHr < 27.5 pg was an optimal cutoff for detecting ID in healthy 9–12- month-old infants. Lorenz et al. recently established a CHr cutoff of <29.0 pg for identifying ID in <32 weeks GA infants at 3–4 months CA (19). Likewise, a Ret-He cutoff of <29 pg has been established for diagnosing ID in very low birth weight preterm infants during the neonatal period (11). Our study demonstrated a Ret-He cut-off of 29.4 or 29.7 pg for identifying ID in <31 weeks GA preterm infants at 4–6 months CA.

Our study used Ret-He instead of CHr, but good agreement has been reported between these two measurements (7). Both CHr and Ret-He measure the amount of hemoglobin in reticulocytes but differ in their measurement methods: CHr uses a two-angle light scatter to measure stained reticulocytes, while Ret-He measures the forward scatter (20). Studies have evaluated the correlation and concordance between Ret-He and CHr, finding them nearly interchangeable for clinical purposes (21).

The spearman correlation coefficient between SF and Ret-He was 0.23, indicating a weak positive relationship between the two metrics. Our findings align with Amin et al.’s study, which also reported a correlation coefficient of 0.21 in similar pediatric cohorts (12). This weak positive relationship may be related to Ret-He and SF reflecting different stages of ID. For instance, Ret-He measures iron in reticulocyte hemoglobin and is insensitive to detecting changes in iron until erythropoiesis is affected (22). In contrast, SF levels decrease in the initial stages of ID when iron stores are first depleted, before other markers like Ret-He and mean corpuscular volume (MCV) become abnormal (23).

Although Ret-He is considered a useful biomarker for assessing iron status in different pediatric populations, its predictive ability to diagnose ID varies considerably across different studies. For instance, Vázquez-López et al. found an AUC of 0.68, similar to ours, for identifying ID in a population of healthy children and adolescents aged 1–16 years (24). However, Neef et al. reported an AUC of 0.77 in a study investigating similarly aged children in a hospital setting (10). Similarly, Davidkova et al. investigated Ret-He’s ability to assess iron status in pediatric chronic dialysis patients ranging from 1 to 18 years of age (9) and reported an AUC of 0.87. The varying results in the latter two studies may be attributed to differences in the patient population since these studies included children with diverse or chronic health conditions. Additionally, the definition of ID differed between the studies. While Vazquez-Lopez had a lower SF cut-off of <10 mcg/L for children aged 1–5 year and <12 mcg/L for children aged 6–16 years for diagnosing ID, the latter two studies defined ID as SF < 100 mcg/L due to the underlying health condition. These differences may indicate that Ret-He measurements only become evident when hemoglobin production is compromised. It is possible that the iron stores in the populations of our study and the study by Vázquez-López et al. were not depleted enough to be detected by Ret-He measurements (22, 24).

Ret-He’s lower predictive ability in our study was similar to Brugnara et al.’s study, which showed relatively low sensitivity (70%) and specificity (78%) of CHr in diagnosing ID in young children (average age: 2.9 years) (17). In contrast, Lorenz et al. (2015) reported a relatively high sensitivity (85%) and AUC (0.86) with specificity of 73% in VPI (<32 weeks GA) at 3–4 months CA. Several factors may contribute to these differences. Firstly, these studies used different definitions for ID. For example, Lorenz et al. defined ID as having two or more of: MCV < 75 fL, transferrin saturation (TSAT) <10%, and SF < 30 ug/L. As can be seen in our study, differences in defining ID led to a different prevalence of ID and contributed to the difference in our AUC and its related properties. Furthermore, our results may differ due to alternate methods for selecting cut-off values. Where our study used Youden’s index to determine the optimal cut-off point, previous studies used methods like selecting cut-offs that favor higher sensitivity or specificity. This is done in circumstances where missing the diagnosis of a disease has serious consequences, and therefore a high sensitivity is favored even if some people are treated without the disease (25). In their study, Lorenz et al. chose their cut-off to achieve a high sensitivity, and accepted that by applying this cut-off, some infants may be treated without having ID, which the authors state was justifiable because iron supplementation does not seem to adversely effect preterm infants in developed countries (26).

Strengths of the study include its population-based design, which allows better representation of the target population and enhances the generalizability of study findings by minimizing bias. The study has limitations, such as its retrospective design, lack of control data from infants born at term, and not including measurements of inflammatory markers. Nonetheless, the study findings based on SF levels can be considered reliable as the subjects were infants who came for growth and neurodevelopmental checks and did not exhibit any acute or ongoing inflammation or infection at the time of follow-up. Another study limitation is a lack of a gold-standard for ID diagnosis. Although SF is currently recommended by the American Academy of Pediatrics for ID diagnosis in children in the absence of inflammation (27), there is evidence to suggest that SF does not correlate well with measures of erythropoiesis in preterm infants beyond 6 months of age (28). Also, the SF levels are not well validated in the preterm population. In the absence of a gold-standard, a combination of tests can probably provide the most accurate diagnostic picture of ID in preterm infant populations. The other most common tests recommended by American Academy of Pediatrics and Swiss Pediatric Hematology Working group for iron status determination are TSAT and serum soluble transferrin receptor. However, they each have limitations. For instance, TSAT is an acute phase reactant and could be affected with infection or inflammation. In addition, serum iron levels, used to calculate TSAT, show diurnal variation and are influenced by oral iron supplements and dietary iron (29). Serum soluble transferrin receptor is limited by high cost, non-standardization of the test and its limited availability in clinical laboratories (6).

## 5. Conclusion

In conclusion, this study suggests that Ret-He is a weak biomarker for detecting ID in former VPI and has low diagnostic accuracy when used in isolation. Therefore, Ret-He should be used in conjunction with measures like SF to improve ID diagnosis. Future studies should continue to investigate optimal approaches for identifying ID in VPI and the clinical utility of Ret-He in combination with other biomarkers.

## Data Availability

The datasets presented in this article are not readily available because they contain information that may compromise the privacy of participants. Requests to access the datasets should be directed to satvinder.ghotra@iwk.nshealth.ca.
